# Mechanical Properties Analysis of Explosive Welded Sheet of AA2519-Ti6Al4V with Interlayer of AA1050 Subjected to Heat-Treatment

**DOI:** 10.3390/ma15114023

**Published:** 2022-06-06

**Authors:** Ireneusz Szachogłuchowicz, Lucjan Śnieżek, Tomasz Ślęzak

**Affiliations:** Faculty of Mechanical Engineering, Institute of Robots & Machine Design, Military University of Technology, ul. gen. S. Kaliskiego 2, 00-908 Warsaw, Poland; lucjan.sniezek@wat.edu.pl (L.Ś.); tomasz.slezak@wat.edu.pl (T.Ś.)

**Keywords:** explosive welding, laminate, AA2519, Ti6Al4V, heat treatment, residual stress measurement

## Abstract

The paper presents results of investigations of welding sheets of AA2519-Ti6Al4V, a difficult-to-joint components materials, produced by explosive welding with a thin technological interlayer of AA1050. The joining process leads to the formation of intermetalics in the vicinity of joint and generates significant residual stresses. In the next step the laminate was subjected to a heat treatment process in order to improve the mechanical properties by precipitation hardening. This treatment should not be carried out before welding because of negative influence on a ductility of the aluminum alloy. Material in this state was subjected to the tests of chemical composition, microstructure, and microhardness. A tensile test was carried out with accompanying strain analysis by the digital image correlation (DIC) method. Moreover, the residual stresses were determined which were measured by using two methods, the X-ray diffraction and the hole drilling. This approach made it possible to measure the residual stresses both in the plane parallel to the surface and in the cross section of the laminate.

## 1. Introduction

Explosive welding technology enable to join the materials difficult-to-join by conventional methods and it is possible to be realized on large plates also. This process is realized by explosively generated pressure wave that imparts very high velocities to the solids (welded materials). During collision the pressure reaches up to 2 × 10^4^ MPa. Such level of pressure makes it possible to obtain physical states unattainable under conventional types of loads [1,2]. As a result, significant residual stresses appear, deviating from the primary distribution in the combined materials [3,4].

AA2519 alloys andTi6Al4V alloys are characterized by increased ballistic resistance. These alloys are used in aerospace industry. The combination of these materials using an explosive welding method should increase ballistic resistance by changing the density centers of the material and the appearance of intermetallics in the joint layer. In the welding zone of Al-Ti explosively produced laminates, the intermetallic phases Al_3_Ti and Ti_3_Al were revealed [5,6,7] which may promote delamination of the produced material and the decrease of strength properties. Additionally, significant residual stresses are present. Therefore, proper heat treatment should be carried out to decrease the negative influence of pointed factors, especially that, the heat treatment is recommended to reduce the adverse effects of the residual stresses formed in the material as a result of the explosive welding process [8]. The most important in such a case is the selection of appropriate processing parameters [9]. In considered laminate, among all components the AA2519 alloy is characterized by the lowest melting temperature, which amounts 821 K. Carefully selected heat treatment parameters, due to the addition of Cu, should provide the effect of precipitation hardening. Additionally, the alloy addition of Zr increases the resistance to secondary recrystallization and increases the tensile strength. It was revealed [10] that the observed intermediate layer is formed by the deposition of aluminum on a titanium substrate due to the interaction of high temperature and pressure. The thickness of this layer depends primarily on the reaction time of the materials at temperature above 873 K. The observed interlayer is formed by mutual diffusion until the formation of Al_3_Ti intermetallic phases. In case of realization of the fusion at the temperature reaching 1073 K, initially Ti_3_Al and TiAl phases are formed. Further maintaining the temperature at 1073 K results in TiAl phases gradually transforming into Ti_3_Al. These transformations lead to the formation of continuous homogeneous Ti_3_Al layer on the titanium surface. In case of Ti_3_Al intermetallic phases, the microhardness can increase up to two times. While the research on microstructure and phase transitions are well described, this study has been undertaken on the influence of heat treatment on the global state of the material.

The purpose of this research is to investigate the influence of hardening heat treatment on the mechanical properties and state residual stresses of explosive welded sheet of AA2519-Ti6Al4V with interlayer of AA1050.

## 2. Materials and Equipment

The subject of the study was an explosive-welded sheet of AA2519-Ti6Al4V with interlayer of AA1050. The AA2519 aluminum alloy is characterized by good fracture toughness because of the copper content of 5.3–6.4%. An important advantage is its increased ballistic resistance and the appropriate weldability. This alloy is strengthened by precipitations through the process of supersaturation and aging. Investigated variety of aluminum alloy was produced by adding the alloying elements Zr and Sc in the amount of 0.1–0.25% for both. The addition of Sc has the effect of fragmenting the structure of the AA2519 alloy, which improves the strength properties of the material. Precipitation strengthening was obtained and the resistance to secondary recrystallization of microstructure increased. The primary effect is a greater stability of mechanical properties at elevated temperature as a result of homogeneous dispersion of Al_3_Zr phase. After casting, the alloy was subjected to hot rolling and then annealing at a temperature of 693 K in air condition for 2 h. A fine-grained structure was obtained, which has a positive effect on the increase of plasticity and fatigue strength. The strength properties and chemical composition of the modified AA2519 alloy are given in Table 1.

The second basic material of the laminate, Ti6Al4V alloy, is used in special constructions which is caused by its high mechanical properties, corrosion resistance, and a melting point reaching 1955 K. For this reason, it is used in the production of aircraft components, including: the jet engine rotor blades or the wing caissons. The strength properties and chemical composition of the Ti6Al4V alloy are presented in Table 2.

Explosive joining of materials with different mechanical properties requires the usage of an interlayer. The AA1050 alloy was used, because it has a very good adhesive property. The strength properties and chemical composition are shown in Table 3.

**Figure 1:**
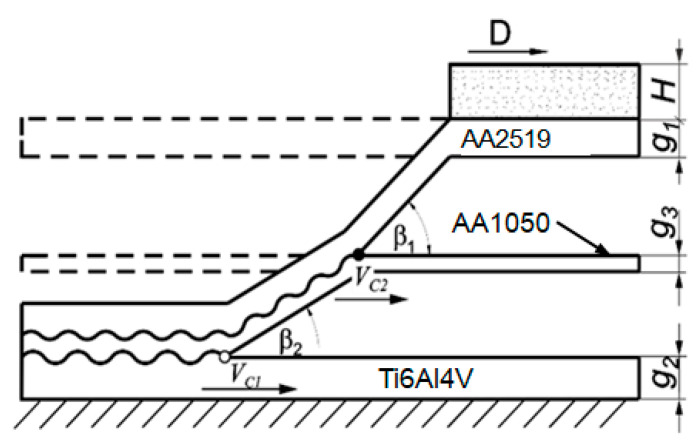
Schematic diagram of the collision course of explosion welded three-layer: β—angle of collision of combined materials, β_1_—angle of collision of base material with interlayer material, β_2_—angle of collision of overlay material with interlayer material V_c_—velocity of collision point with respect to contact point of welded plates, V_c1_—velocity of collision point of contact between base material and interlayer material, V_c2_—velocity of the collision point of the contact point of the base material and the interlayer, D—velocity of detonation of the explosive, H—height of the explosive layer, g_1_—thickness of the plate to be shot, g_2_-thickness of the base plate, g_3_—thickness of the interlayer.

The layered material subjected to testing was manufactured in the Department of High Energy Technologies EXPLOMET Galka Szulc general partnership. The functional properties of explosives, including the detonation velocity, are derived from their chemical composition, the physical form of their individual components, e.g., fragmentation, the degree of pore surface development present in the structure of crystals of the ammonium nitrate used, and the quality of their mixing. The assumed plan of plating tests included the execution of joints in the AA2519-Ti6Al4V double-layer system with a separate spacer made of AA1050 aluminum alloy (Figure 1) [11].

**Figure 2:**
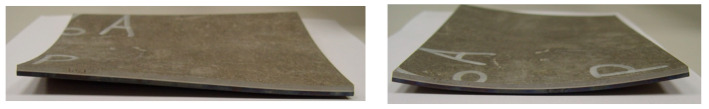
Explosive welded sheet of AA2519-Ti6Al4V with interlayer of AA1050 after heat treatment.

The final products were in the form of plates with dimensions of 200 × 150 mm and thickness of 6.7 mm, approximately (Figure 2). The thickness can slightly vary because of the dynamic nature of the joining process. The plates were rolled in order to give them adequate flatness. Both described technological processes significantly deformed the material and introduced elastic-plastic residual strains. In the next step the plates were annealed at the temperature of 803–823 K for 2 h, cooled down in water at room temperature and aged at 438 K for 10 h. The main aim of this treatment was to obtain an effect of precipitation hardening. Besides the changes of microstructure and mechanical properties this treatment has seriously influenced the residual stresses state.

**Figure 3:**
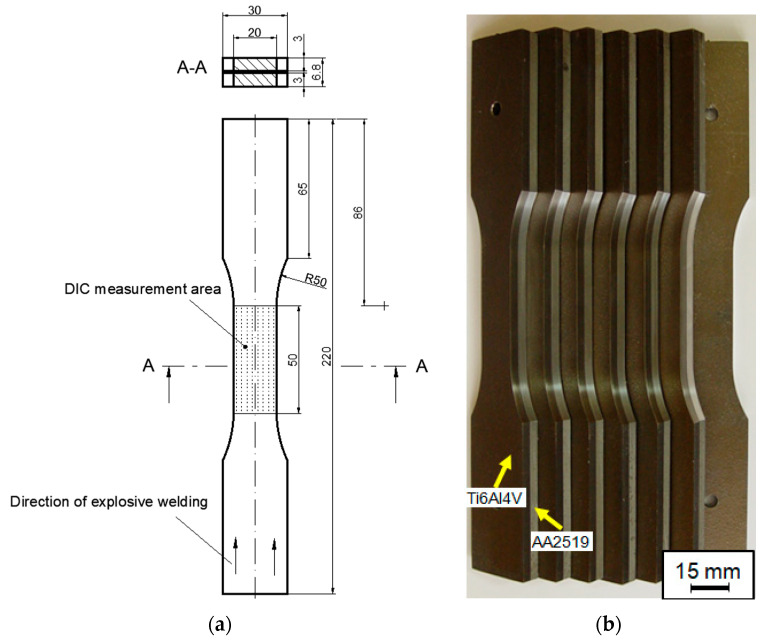
Dimensions of samples for tensile testing of AA2519-Ti6Al4V laminate with inter-layer of AA1050 (**a**) and prepared test specimens (**b**).

Monotonic tensile tests were carried out on specimens made of manufactured laminate after heat treatment. All the specimens subjected to axial tension had the same geometry. Five specimens were made of each material (Figure 3). Tensile testing of AA2519-Ti6Al4V laminate with AA1050 interlayer in axial tension conditions was performed according to PN-EN ISO 6892-1:2010 on an Instron 8802 monotonic and fatigue hydraulic pulser.

Microhardness tests were carried out using a fully automatic universal laboratory hardness tester DURA SCAN 70. The microhardness test was conducted in accordance with EN ISO 6507-1. The indenter load was F = 0.9807 N. The process time to load the indenter to the nominal load was 3 s. It took 10 s to load the sample to 0.98 N.

**Figure 4:**
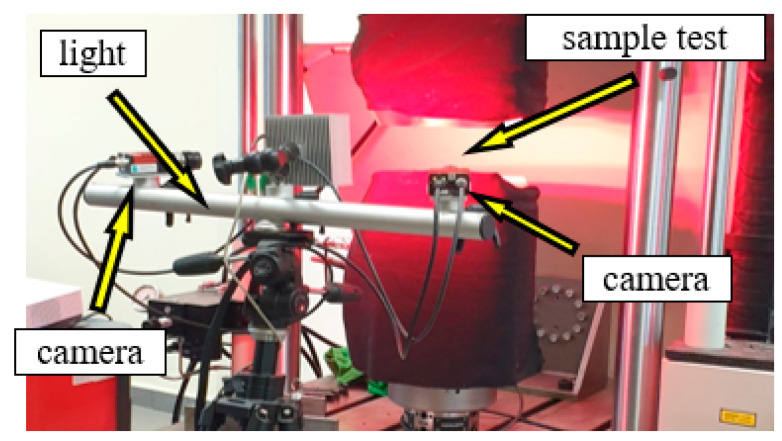
Digital image correlation measurement system from Dantec.

Digital image correlation (DIC) allows the measurement of deformations and deformation of the surface of the tested object (Figure 4). The digital image correlation method is used in optical, non-contact, and three-dimensional systems for real-time measurement of displacement and deformation. The DIC system allows speckle images to be captured using CCD cameras, while further data analysis and processing are carried out using Istra 4D software.

**Figure 5:**
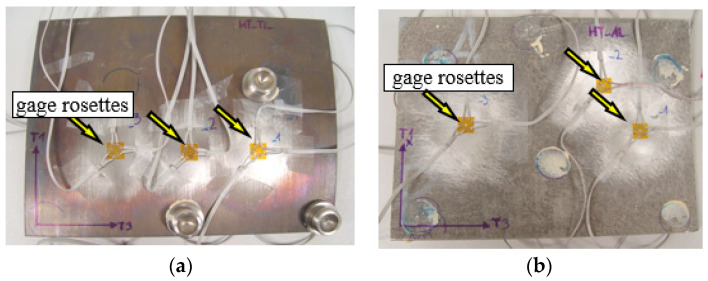
Arrangement of gauge rosettes on TiAl4V side 9 (**a**) AA2519 (**b**).

The procedure for measuring residual stresses using the hole trepanation method is standardized by ASTM Standard Test Method E 837 [12], and the technical procedure of implementation is described in Tech Note TN-503 [13]. Micro-Measurement RS-200 milling guide was used to conduct the hole drilling process, necessary in order to measure the residual stresses. This equipment enables precise drilling of holes in the center point of strain gauge rosette which is the most important condition for making correct measurements. Figure 5 shows the distribution of gauge rosettes.

**Figure 6:**
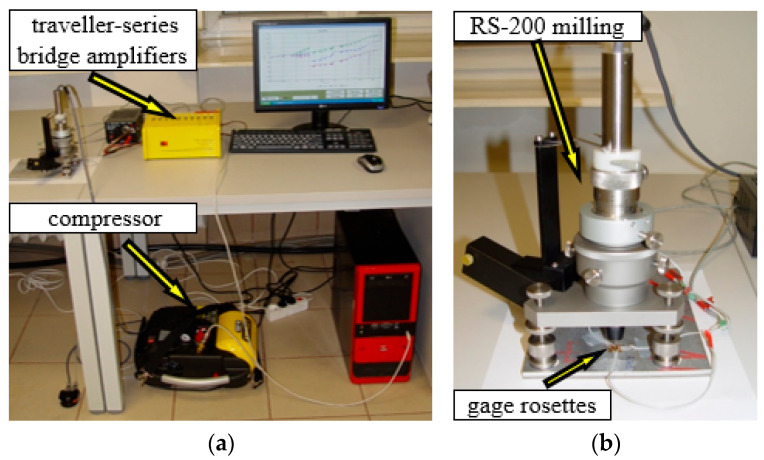
The overall view of testing stand (**a**) and the milling guide of RS-200 (**b**).

Furthermore, it enables to control the depth of hole and to measure its diameter. The carbide milling cutters with a diameter of 1.6 mm were used during the tests and these tools were driven by high-speed rotation air turbine supplied by compressor. Testing stand is presented in Figure 6.

**Figure 7:**
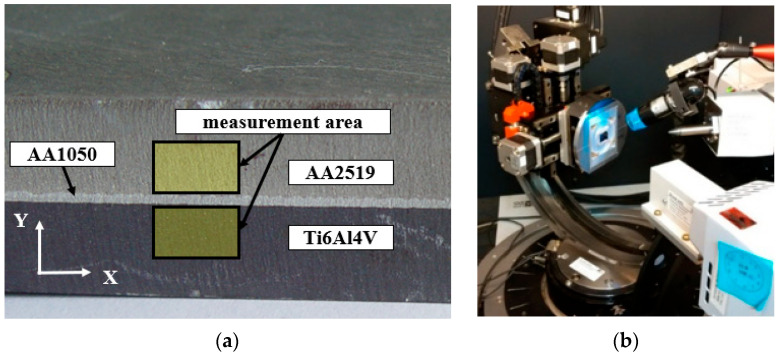
The surface area of the sample (**a**) and Bruker D8 Discover diffractometer with Euler wheel (**b**).

Surface residual stresses in the two main, perpendicularly oriented directions (“σ1, σ2”) based on sin2ψ diffractometric measurements were obtained using an X-ray diffractometer -Bruker D8 Discover (Bruker Corporation, 40 Manning Road, Billerica, Massachusetts, United States of America) with a Euler wheel and a sample positioning system along the three axes. Test samples for the research were prepared using electrical discharge machining. Radiation and beam optics were characterized by CoKα filtration. Phase analysis was performed in the Crystal Impact Match software with an ICDD PDF 4+ 2019 crystal-lographic database. TARSIuS (Texture-Aided Residual Stress Investigation System) pack-age is a software developed by the Institute of Metallurgy and Materials Sciences of the Polish Academy of Sciences in Krakow and it is used to visualize the residual stresses in materials. Because this method is quite new, we decided to implement it in a short explanation of its basics with examples. The areas were near to the connection of the plates and the detailed locations are shown in the Figure 7.

## 3. Results

**Figure 8:**
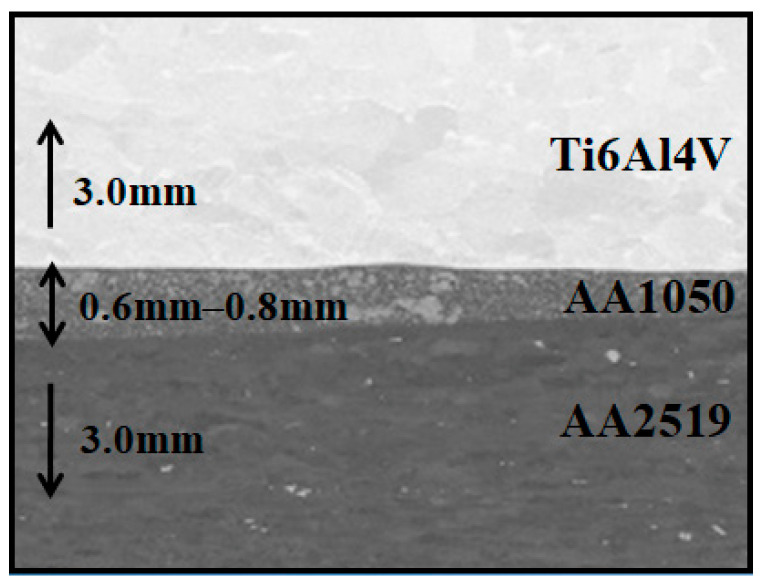
Explosive welded sheet of AA2519–Ti6Al4V with interlayer of AA1050) a metallographic cross-section of the composite.

In the Figure 8 the view of plates after heat treatment and the metallographic cross-section of the composite are shown.

The picture in the Figure 1 has shown that the plates after heat treatment are significantly distorted. This deformation is caused by the difference of the value of the coefficient of thermal expansion and the characteristic temperatures of phase transitions.

**Figure 9:**
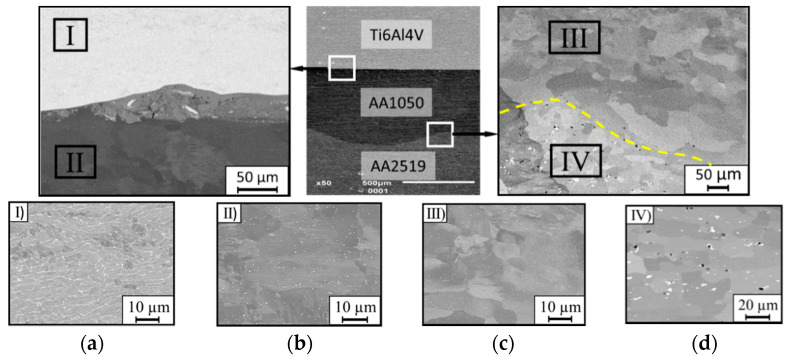
Metallographic cross-section of the produced explosive welded sheet of AA2519-Ti6Al4V with interlayer of AA1050 after heat treatment: (**a**) sector I–Ti6Al4V alloy; (**b**) sector II–AA1050; (**c**) sector III–AA1050; (**d**) sector IV–AA2519 alloy.

The microstructure of the heat treated of explosive welded sheet of AA2519-Ti6Al4V with interlayer of AA1050 in the vicinity of joint is shown in the photographs (Figure 9).

Heat treatment has not affected the structure of titanium alloy (Figure 9a). The AA1050 interlayer by the boundary with titanium is characterized by the grain growth with uniform distribution (Figure 9b). Near the AA2519 alloy, the grains of the AA1050 alloy structure have an equiaxial character (Figure 9c). For AA2519 alloy, the heat treatment process improved the solubility of copper-rich precipitates in the aluminum matrix. The effect of this process is the fine-grained structure and steady distribution of precipitates in the matrix. At the interface between AA1050 aluminum and AA2519 alloy, a sublayer of about 4–6 µm width was observed, which was formed as a result of heat treatment. It is characterized by a fragmented structure and numerous voids with diameters of about 0.3–0.5 µm (Figure 9d). The slight voids were probably produced accidentally during the supersaturation and annealing process.

### 3.1. Strength Properties

**Figure 10:**
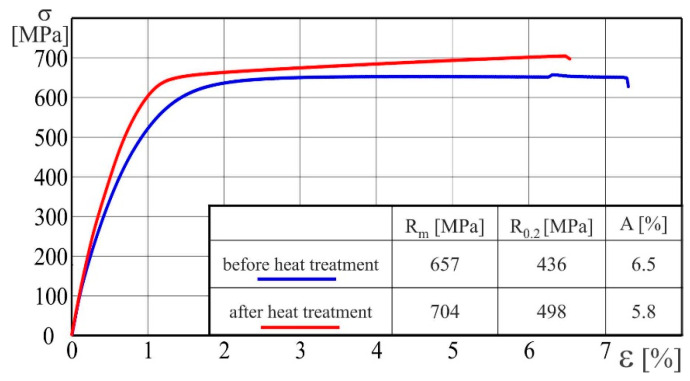
Stress–strain curves of AA2519-Ti6Al4V laminate with interlayer of AA1050 in production state and after heat treatment.

Tensile tests of AA2519-Ti6Al4V laminate with interlayer of AA1050 were conducted according to [14]. The tests were carried out using five samples and obtained results were repeatable. Samples were cut along the direction of explosive welding. The results obtained during the tests are presented in the form of graphs in Figure 10.

The heat treatment has resulted in an increase of the ultimate strength Rm from 657 MPa up to 704 MPa, which is about 7%, whereas the yield strength R_0.2_ has increased from 436 MPa to 498 MPa and the increase is about 14%. On the other hand, the heat treatment has negative influence on the elongation, A, which has decreased from 6.5% to 5.8%. Based on these results, it can be stated that the ductile of heat-treated laminate decreased.

**Figure 11:**
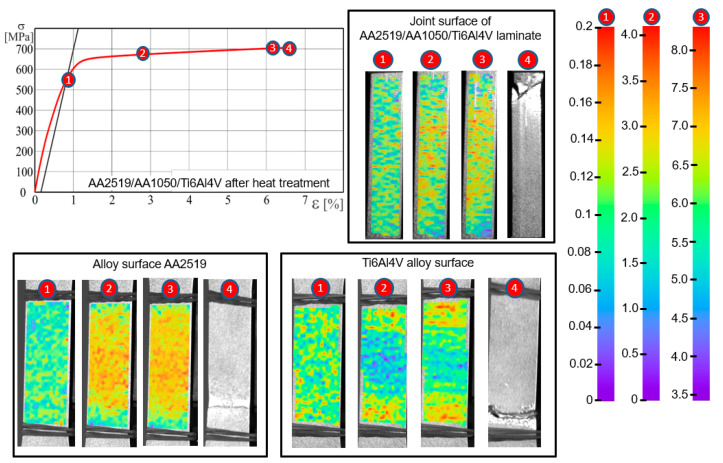
Strain distribution using DIC method for selected points of the stress-strain curve obtained for AA2519-Ti6Al4V laminate with interlayer of AA1050 after heat treatment: 1—The yield strength R0.2; 2—the plastic strain of 2%; 3—the ultimate strength Rm; 4—the fracture of the specimen.

During tensile tests, an analysis of strain distribution using was applied using digital image correlation (DIC). In case of the laminate subjected to additional heat treatment, the strain distribution for the AA2519 alloy, up to the moment of a sample rupture, is characterized by high uniformity (Figure 11).

In case of the Ti6Al4V alloy, after exceeding the yield point, the strain maps illustrate the occurrence of horizontal bands. DIC analysis made on the lateral surface has not revealed local or banded strain inhomogeneity, which makes it impossible to predict the location of cracking even under high loadings.

### 3.2. Microhardness

**Figure 12:**
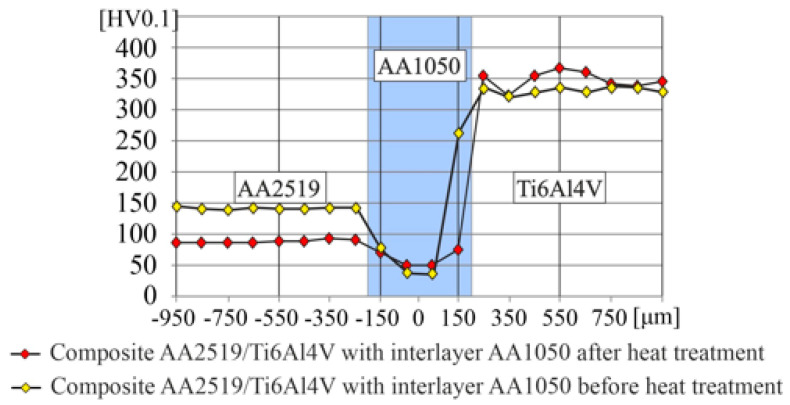
The results of microhardness measured in AA2519–Ti6Al4V laminate with interlayer of AA1050.

The measurement of microhardness HV0.1 was conducted according to the standard [15]. The study was performed in the immediate vicinity of the center layer. Obtained results are presented in the Figure 12, where the AA1050 intermediate layer is in grey.

The microhardness of the AA2519 alloy after heat-treatment was below 100 HV. In case of the interlayer, which is composed of the AA1050 alloy, there is noticeable a strengthening effect near the boundary with the AA2519 due to the formation of intermetallic precipitates. In case of Ti6Al4V alloy, the microhardness after heat treatment was about 350 HV and has not changed compared to the state before heat treatment [16].

### 3.3. Measurements of Residual Stresses

The applied processes of explosive joining and following rolling significantly influenced the stress state of the material. It is important to define this state, therefore the residual stresses were measured in two perpendicular planes, namely in the plane parallel to the surface and in the cross section. The first measurements were conducted using hole-drilling method and the second by X-ray diffraction.

#### 3.3.1. Hole-Drilling Method

This method was used to determine the state of surface residual stresses and their change with the thickness. The measurements were made at three randomly placed points on both sides of the plate, from the aluminum and titanium. In the presence of residual stresses, even a small hole into the material causes stress relaxation because each normal stress component perpendicular to the free surface (e.g., the hole surface) is zero. Moreover, this relaxation is related to the change in strain field in the direct vicinity of the hole according to Hook’s law. This method is often referred to as a “semi-destructive” technique because in many cases a small hole does not significantly damage the structural integrity of the test object (the dimensions of the hole are usually 0.8–4.8 mm-diameter and depth). After measuring large objects, it is possible to remove the hole by careful welding and smoothing the surface with a grinder.

**Figure 13:**
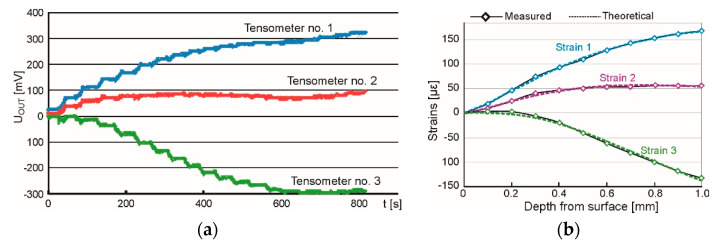
The overall view of testing stand (**a**) and the milling guide of RS–200 (**b**).

The obtained output voltage was transmitted from the rosette to the channels of the ESAM Traveler Plus strain gauge bridge, where it was amplified. An exemplary graph presenting the changes of voltage recorded during the test is presented in Figure 13a.

The calculation of the strain values was made up to the depth of 1.0 mm employing Equation (1).

Ε = 4 × U_OUT_ / (U_0_ × N × K × A) × 10^6^ [µε = 10^−6^] (1)

where:

U_OUT_—output voltage [V];
U_0_—supply voltage [V];
N—coefficient depending on the bridge type—for quarter-bridge N = 1;
K—strain gauge constant;
A—coefficient of output signal amplification.

As a result of the measurements and calculations carried out, the characteristics of changes in relative strain values as a function of the depth of the hole drilled were obtained. To determine the values of principal stresses and their direction, the following relations were applied.

(2) $$\sigma_{max}=\frac{\varepsilon_{1}+\varepsilon_{2}}{4\cdot A}-\frac{1}{4\cdot B}\sqrt{{\left(\varepsilon_{3}-\varepsilon_{1}\right)}^{2}+{\left(\varepsilon_{3}+\varepsilon_{1}-2\varepsilon_{2}\right)}^{2}\;}\left[\mathrm{MPa}\right]$$

(3) $$\sigma_{max}=\frac{\varepsilon_{1}+\varepsilon_{2}}{4\cdot A}+\frac{1}{4\cdot B}\sqrt{{\left(\varepsilon_{3}-\varepsilon_{1}\right)}^{2}+{\left(\varepsilon_{3}+\varepsilon_{1}-2\varepsilon_{2}\right)}^{2}\;}\left[\mathrm{MPa}\right]$$

(4) $$\alpha =\frac{1}{2}arctg\frac{\varepsilon_{1}-2\varepsilon_{2}+\varepsilon_{2}}{\varepsilon_{2}-\varepsilon_{1}}\left[\mathrm{MPa}\right]$$

where:

*σ_max_*, *σ_min_*—principal stresses;
*ε*_1_, *ε*_2_, *ε*_3_—strains measured on strain gauges number 1, 2, and 3;
*A*, *B*—coefficients depending on material properties and geometry of rosette and hole;
*α*—angle between strain gauge no. 1 and the direction of the nearest principal stress.

The result of performed analysis is presented in Figure 13b. Calculated changes of strains (dashed lines) correspond to the measured data (diamonds). This accordance was gained under the assumption that the residual stresses vary linearly with the depth. Determination of the stress value and its character was accomplished using software H-Drill. Obtained results of measured principal residual stresses and the angle from the principal axis to defined direction, are presented in Table 4. The minus value of the angle indicates that the angular deviation is counterclockwise.

The character of stresses was analyzed up to the depth of 1.0 mm and in all cases a linear character of the stress changes was revealed. This assumption was justified by the values of statistical parameter RMS misfit which vary from 1.9 me to 2.8 me for AA2519 and from 4.6 me to 6.0 me for Ti6Al4V. If the uniform state of stresses was assumed, the RMS misfit would have vary from 6.8 me to 11.6 me and from 9.1 me to 16.2 me, respectively.

Determined values of principal stresses in aluminum alloy AA2519 vary with the depth within the ranges: σ_min_ from −102 MPa up to +90 MPa and σ_max_ from −27 MPa up to +146 MPa. On the surface, the values of σ_min_ and σ_max_ are −96.0 ± 5.9 MPa and −20.7 ± 7.6 MPa, respectively. The stresses are compressive and at different depths change to tensile stresses. Comparing the principal directions of residual stresses with the geometry of investigated plate it can be stated that σ_min_ is oriented perpendicular to the distortion, namely parallelly to the maximum gradient, and σ_max_ opposite. The values of principle stresses in titanium layer change with the ranges: σ_min_ from +8 MPa up to +376 MPa and σ_max_ from +132 MPa up to +509 MPa and they were determined at different depths. The residual stresses are tensile and increase linearly for the surface values of σ_min_ and σ_max_ equal to +36.0 ± 21.7 MPa and +141.3 ± 11.8 MPa, respectively. It must be pointed that the maximum values determined at greater depths can be subjected to high uncertainty. When drilling a hole in the titanium alloy, high cutting resistance was observed which may influence the closest vicinity of the hole, thus the near-surface values of residual stresses are most reliable. The directions of principal stresses are oriented similarly to previously described but σ_min_ and σ_max_ are swapped because on this side of the laminate the surface is convex (on the side of aluminum is concave) which changes the compressive stresses to tensile.

#### 3.3.2. X-ray Diffraction Method

**Figure 14:**
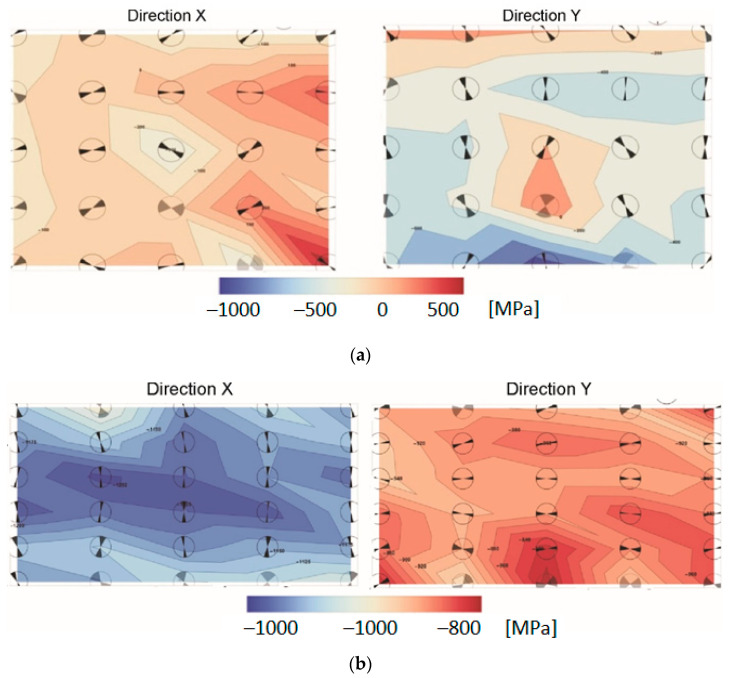
The maps of residual stresses measured in two perpendicular directions X and Y in the (**a**) AA2519 alloy and (**b**) Ti6Al4V; description in text.

Based on the gained data, the two-dimensional maps of residual stresses were developed whose visualization enable easy interpretation of the stress state (Figure 14).

Determined values of residual stresses measured in the vicinity of the joint show that the directions of principal stresses are oriented approximately parallelly/perpendicularly to this joint. It is indicated by the orientation of the angular marks in the circles placed at the measuring points (Figure 14). Residual stresses are both compressive and tensile in AA2519, but in Ti6Al4V are only compressive. The measurement results presented in form of maps are the most reliable for the titanium alloy layer. The maximum residual is in its central part, where they reach the value of −1250 MPa. The lower values of compressive stresses in the horizontal direction may be related to the change of shape of the plate after heat treatment, which is slightly distorted.

The values of stresses in the layer of AA2519 alloy should be treated as speculative, because the values significantly exceeded the yield point of this material. This is most likely due to the formation of intermetallic phases in the aluminum and grain growth.

## 4. Summary and Conclusions

The results of measurements the residual stresses obtained by two different methods seem to be inconsistent with each other thus this issue needs wider explanation in order to clarify doubts. First difference relates to the orientation of the measuring plane—the hole-drilling method (HDM) was applied in plane parallel to the surface contrary to X-ray method (XRM), which was applied in perpendicular plane. Second difference is related with the place of measurements and state of the material. HDM has been adopted to determine the stresses on the surface and at shallow depth of the material in the form of full-scale plate after heat treatment without any additional mechanical processing. Whereas XRM allowed to measure the stress state near the interlayer, but the small sample must be cut from the plate for proper preparation. This process can influence the global stress state. The last difference relates to the nature of the measurement. HDM allows to determine only the value of the first-order residual stresses because the resultant strains are measured in some distance from the measurement point. XRM is based on the measurement of the distance between the crystallographic planes thus the values can be connected with the microscale second- or even third-order residual stresses and the microscale mechanical properties. The influence of this problem was observed during the measurement of stresses in aluminum alloy when the determined values significantly overcame the macroscale tensile strength. The above-described differences between HDM and XRM indicate that comparisons of results should be made with caution.

The investigation was performed to analyze the influence of the heat treatment on the structure and the mechanical properties of the AA2519-Ti6Al4V laminate with interlayer of AA1050. The main purpose of the heat treatment was to obtain an effect of precipitation hardening in aluminum alloy, which was successfully gained. Nevertheless, this process has seriously influenced the residual stresses state. The laminate was distorted because of the differences in thermophysical properties thus there were performed wide investigation on the state of residual stresses. On the basis of the obtained results the following conclusions can be stated:

1. A heat treatment affects the laminate resulting in an increase of the ultimate strength Rm about 7% to the value of 704 MPa and the yield strength R_0.2_ about 14% to the value of 498 MPa, but it has also negative influence on the ductility decreasing an elongation from 6.5% to 5.8%;
2. As a result of heat treatment, the microhardness of titanium alloy remains unchanged but in AA2519 it increased from app. 95 HV_0.1_ [13] to the value of 150 HV_0.1_;
3. The heat treatment in AA2519 caused fine-grained structure with steady distribution of precipitates in the matrix and additionally a slight sublayer of about 4–6 µm width with some voids observed at the interface between this material and interlayer;
4. The surface residual stresses were determined by the hole drilling method and are closely related to distortion. They are compressive on the side of aluminum alloy (to −102 MPa) and tensile on the side of titanium alloy (up to +158 MPa);
5. The residual stress state in cross-section was determined by X-ray diffraction method. They are compressive reaching −1250 MPa in Ti6Al4V. The results for AA2519 are very dubious.

## Figures and Tables

**Table 1:** Strength properties and chemical composition of the AA2519 alloy.

Strength Properties			Chemical Composition [wt%]									
R_0.2_ [MPa]	R_m_ [MPa]	A [%]	Si	Fe	Cu	Mg	Zn	Ti	V	Zr	Sc	Al
312	335	6.5	0.06	0.08	5.77	0.18	0.01	0.04	0.12	0.2	0.36	rest

**Table 2:** Strength properties and chemical composition of the Ti6Al4V alloy.

Strength Properties			Chemical Composition [wt%]							
R_0.2_ [MPa]	R_m_ [MPa]	A [%]	O	V	Al	Fe	H	C	N	Ti
950	1020	14	<0.20	3.5	5.5	<0.30	<0.0015	<0.08	<0.05	rest

**Table 3:** Strength properties and chemical composition of the AA1050 alloy.

Scheme			Chemical Composition [wt%]							
R_0.2_ [MPa]	R_m_ [MPa]	A [%]	Fe	Si	Zn	Mg	Ti	Mn	Cu	Al
78	168	2.9	0.4%	<0.25	<0.07	0.18	<0.05	<0.05	<0.05	rest

**Table 4:** The results of residual stresses measurements of the heat treated laminate AA2519-Ti6Al4V laminate with interlayer of AA1050.

	AA2519								
	Number of Measuring Point								
	# 1			# 2			# 3		
depth [mm]	0.0	0.5	1.0	0.0	0.5	1.0	0.0	0.5	1.0
	residual stresses s [MPa] and angular orientation a [°]								
s_max_	−10	49	108	−27	59	146	−25	56	136
s_min_	−102	−66	−31	−98	−26	45	−88	1	90
a	−81	−77	−74	−88	−82	−77	−86	−86	−87
	**Ti6Al4V**								
	**Number of measuring point**								
	**# 1**			**# 2**			**# 3**		
depth [mm]	0.0	0.5	1.0	0.0	0.5	1.0	0.0	0.5	1.0
	residual stresses s [MPa] and angular orientation a [°]								
s_max_	134	285	436	132	166	201	158	329	509
s_min_	8	120	232	39	85	128	61	223	376
a	−32	−29	−28	−19	−25	−31	−6	7	17

## Data Availability

Not applicable.
